# Supplementary figures and images for: A Systemic View of Carbohydrate Metabolism in Rice to Facilitate Productivity
Source: Plants (Basel). 2021 Aug 17;10(8):1690. doi: 10.3390/plants10081690 (PMC8401045; doi:10.3390/plants10081690)

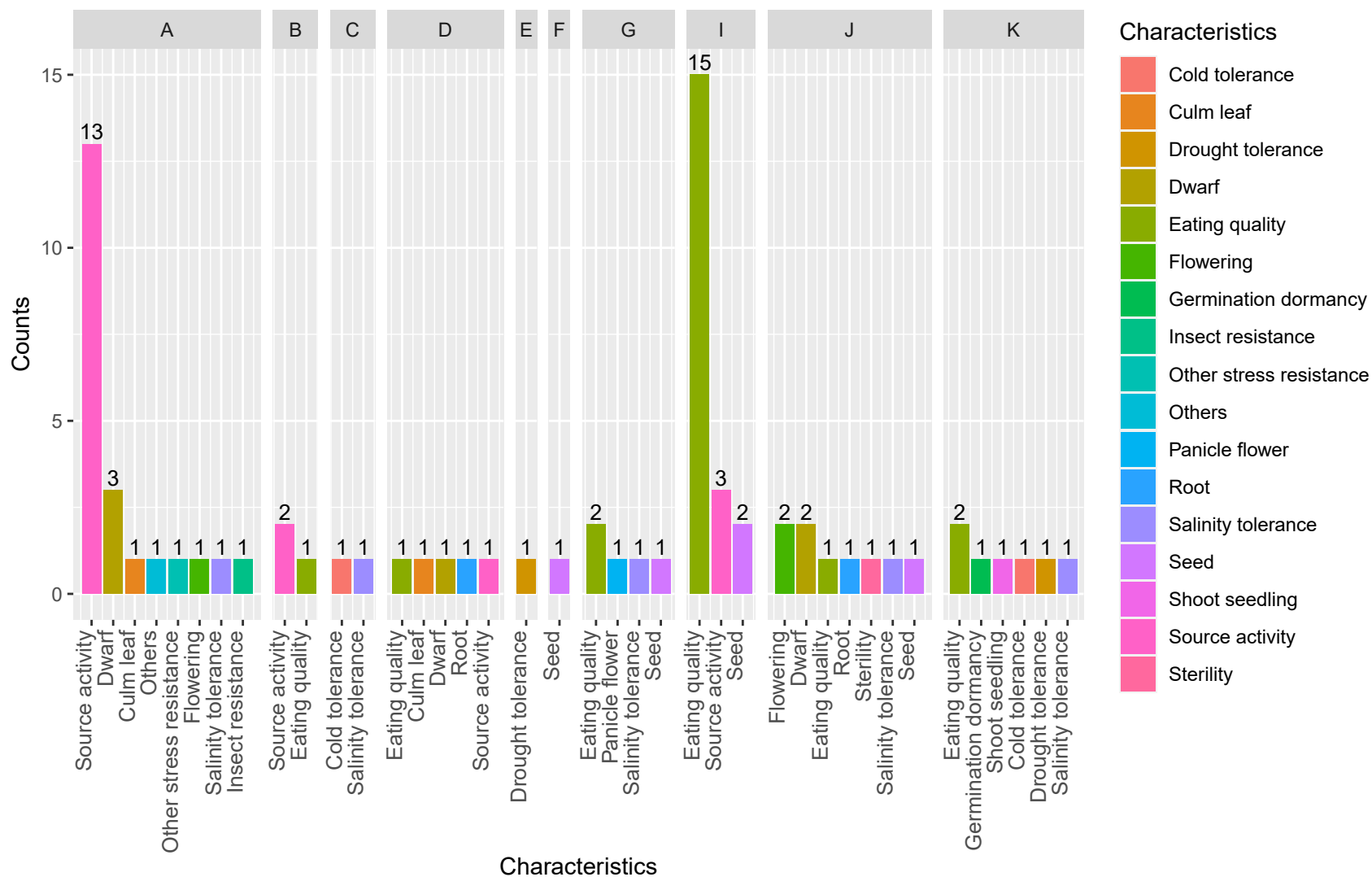

Supplement: Supplementary file 1 [file plants-10-01690-s001.zip › plants-1321669-supplementary/Figure S1.pdf]
